# Supplementary material for: Salmonirosea aquatica gen. nov., sp. nov., a Novel Genus within the Family Spirosomaceae, Was Isolated from Brackish Water in the Republic of Korea
Source: Microorganisms. 2024 Aug 14;12(8):1671. doi: 10.3390/microorganisms12081671 (PMC11356934; doi:10.3390/microorganisms12081671)
Supplement: Supplementary file 1 [file microorganisms-12-01671-s001.zip › microorganisms-3049619-supplementary.pdf]

Supplementary Material

Salmonirosea aquatica gen. nov., sp. nov., a Novel Genus within the Family *Spirosomaceae*, was Isolated from Brackish Water in the Republic of Korea

Kiwoon Baek, Sumin Jang, Jaeduk Goh and Ahyoung Choi*

Biological Resources Research Department, Nakdonggang National Institute of Biological Resources (NNIBR), Sangju 37242, Republic of Korea

* Correspondence: aychoi@nnibr.re.kr; Tel.: +82-54-530-0891; Fax: +82-54-530-0899

Table S1. Summary of the complete genome sequence of strain SJW1-29^T. Information on the genome sequencing project is available under the accession number WHLY00000000 at the assembly in NCBI.

**Features**	**SJW1-29**
Sequencing platform	PacBio RS II
Library used	SMRTbell library
Sequencing coverage	93.83 ×
Assembler	PacBio SMRT Analysis 2.3.0
Genome size (bp)	7,065,248
N50 (bp)	6,512,965
No. of CDSs	6,297
No. of tRNA genes	40
No. of rRNA genes	9
Number of contigs	4

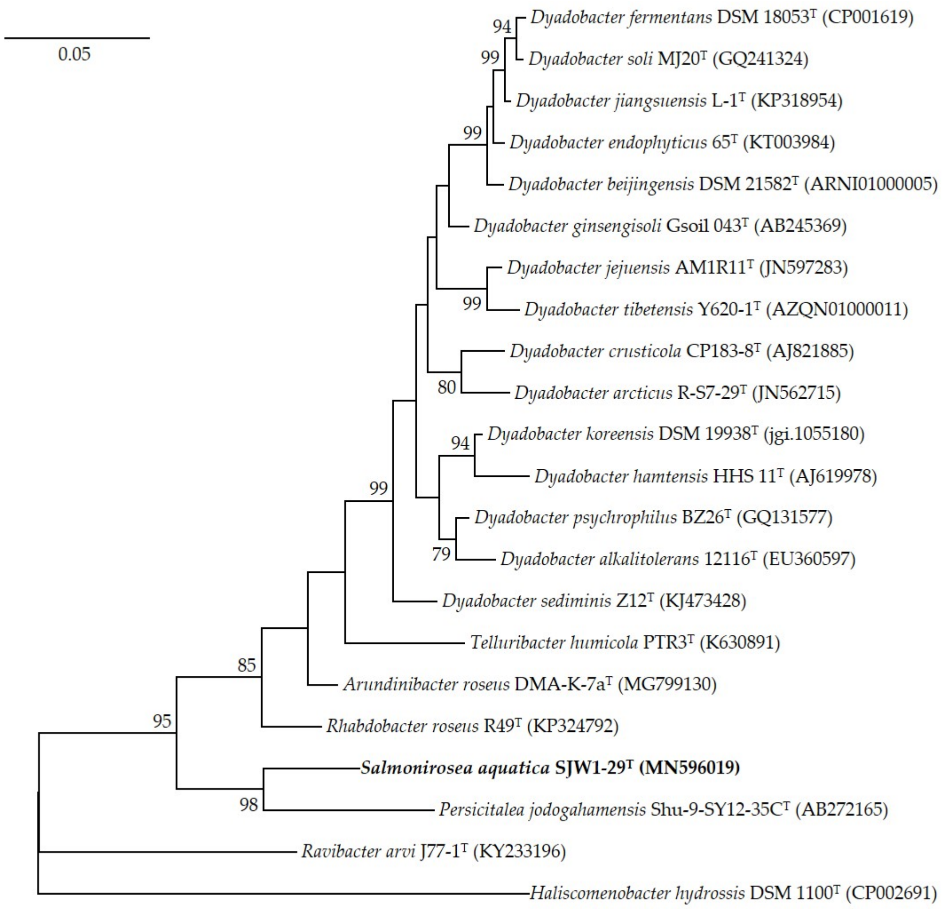

Figure S1. Maximum-likelihood tree based on nearly complete 16S rRNA gene sequences showing the phylogenetic position of strain SJW1-29^T with closely related taxa. Bootstrap values >70 are indicated and based on 1,000 replications. *Haliscomenobacter hydrossis* DSM 1100^T (CP002691) was used as an out-group. The scale bar depicts 0.05 substitutions per nucleotide position.

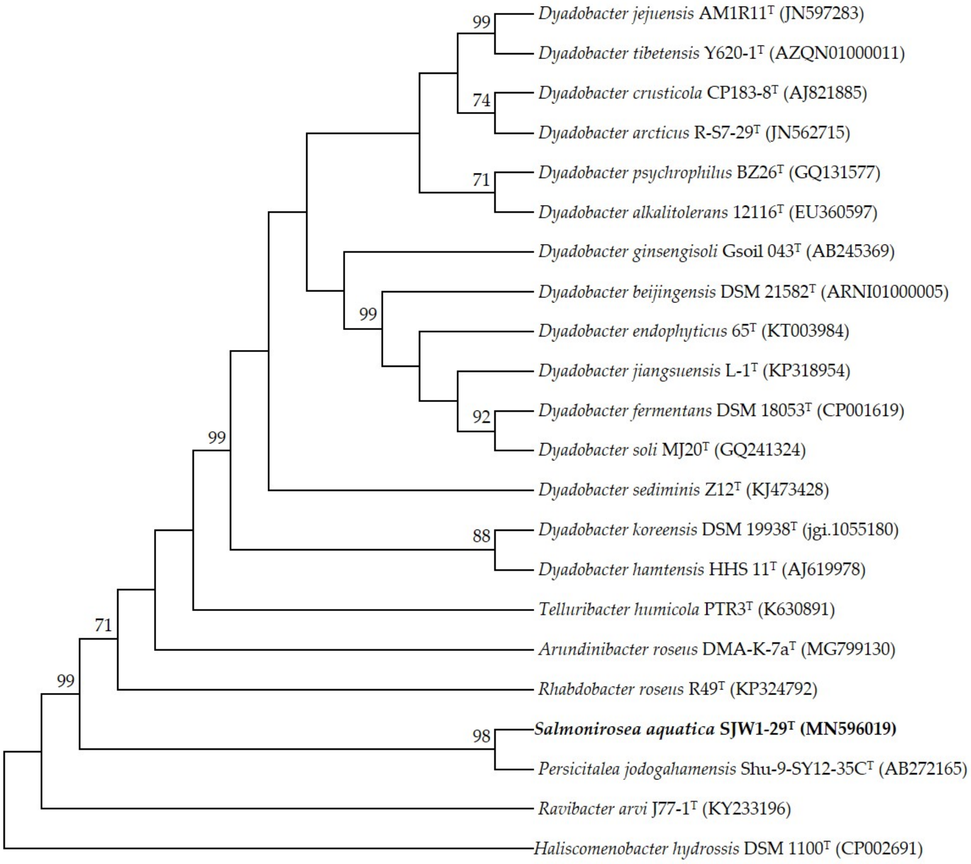

Figure S2. Maximum-parsimony tree based on nearly complete 16S rRNA gene sequences showing the phylogenetic position of strain SJW1-29^T with closely related taxa. Bootstrap values >70 are indicated and based on 1,000 replications. *Haliscomenobacter hydrossis* DSM 1100^T (CP002691) was used as an out-group.

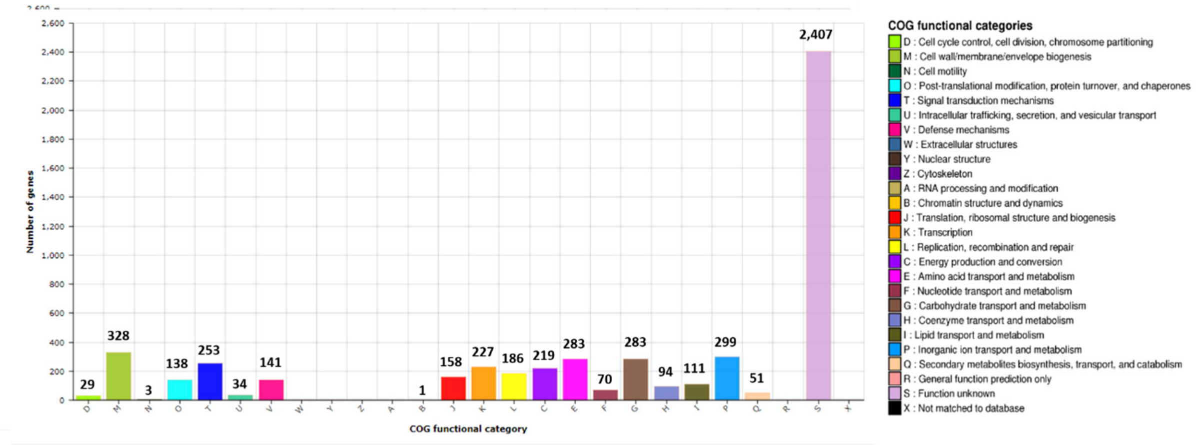

Figure S3. COG functional classification of proteins in strain SJW1-29^T genome.

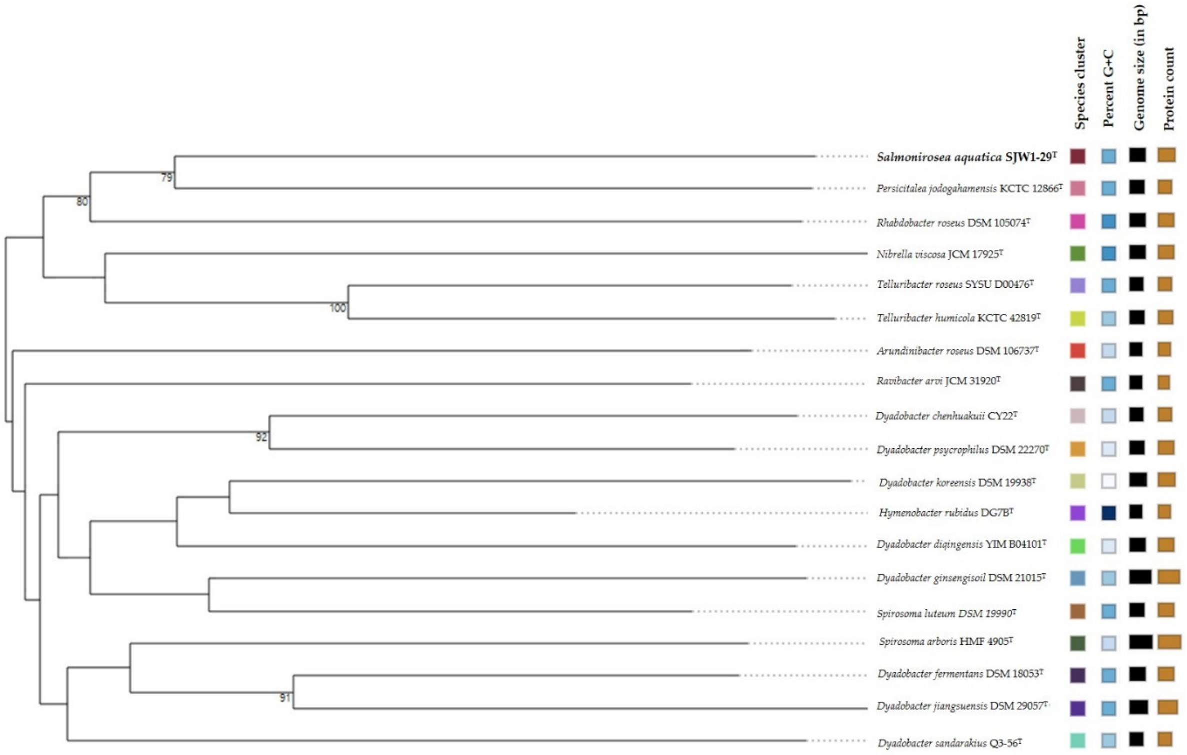

Figure S4. Whole-Genome sequence-based phylogenetic tree of strains SJW1-29T using TYGS design tree with FastME 2.1.4 software based on Genome BLAST Distance Phylogeny (GBDP) parameters. Distances were calculated from the genome sequences, and branch lengths were calculated by GBDP distance formula d5.

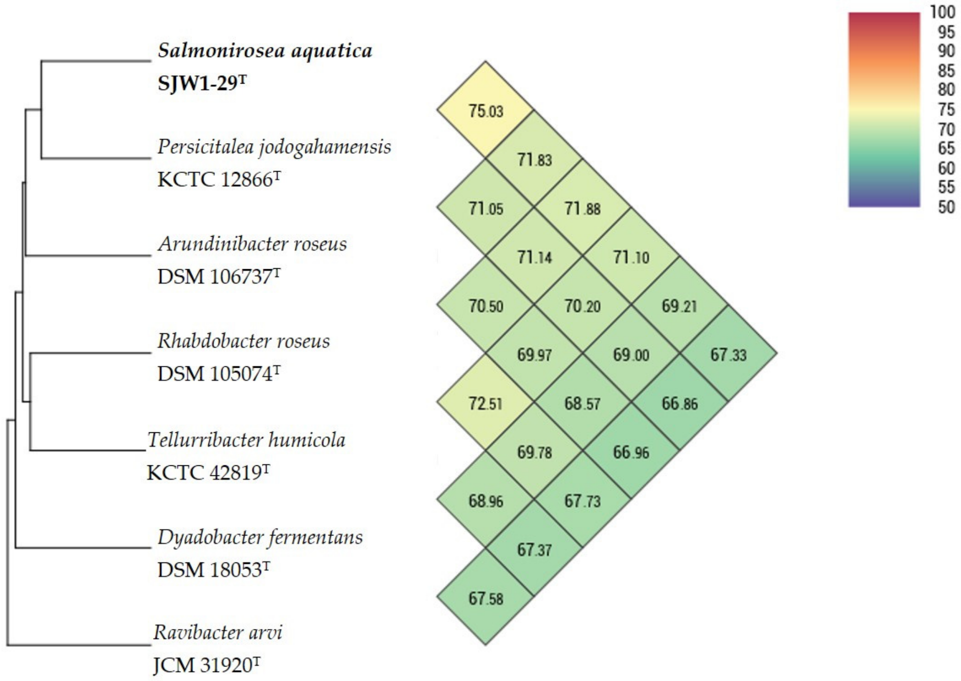

Figure S5. Heatmap of Orthologous Average Nucleotide Identity (OrthoANI) between strain SJW1-29T

and closely related relatives. Values in the color scale indicate the similarity percentage among the genomes. The numerical values on the branches represent the calculated intergenomic genetic distance calculated by the Orthologous Average Nucleotide Identity Tool (OAT) software.

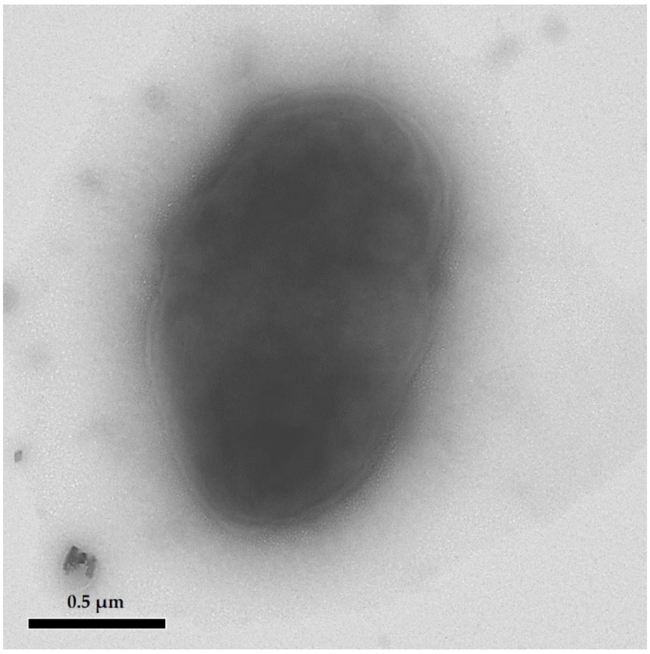

Figure S6. Transmission electron micrograph of SJW1-29^T cells. Bar, 0.5 μm.

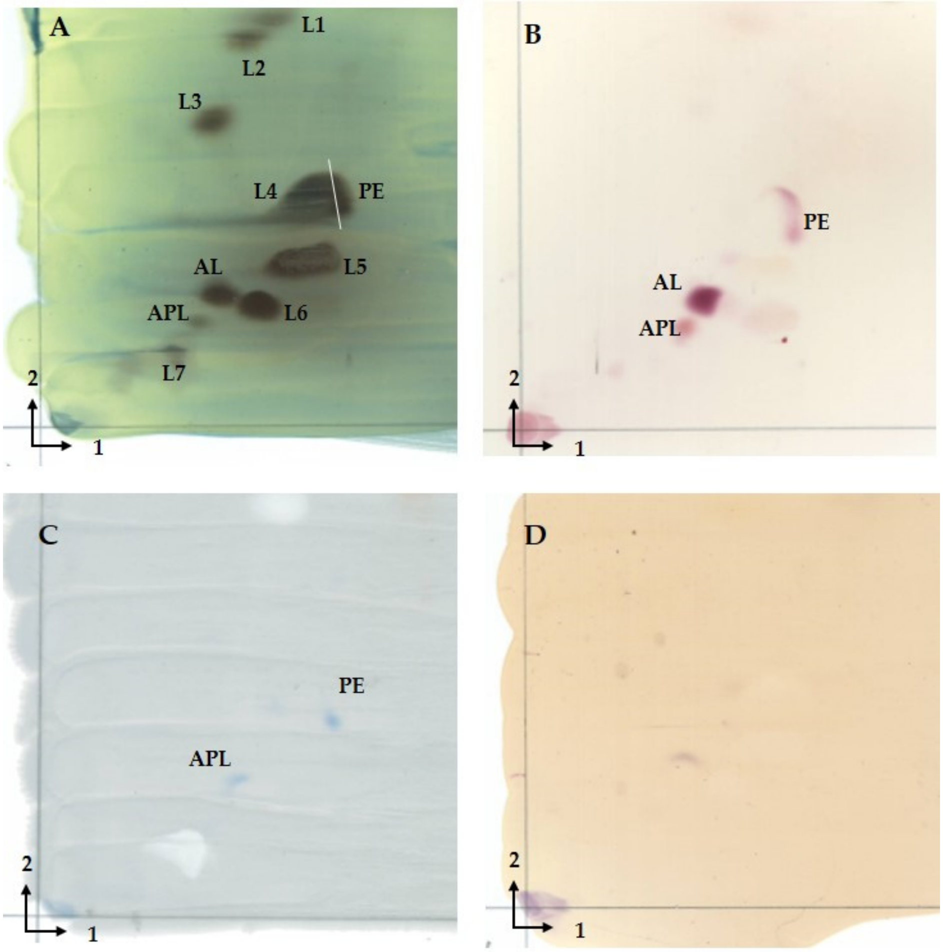

Figure S7. Two-dimensional thin-layer chromatogram showing the polar lipids of SJW1-29^{T}. Chloroform/methanol/water (65:25:4) was used in the first dimension and chloroform/acetic acid/methanol/water (40:7.5:6:2) was used in the second dimension. The following spray reagents were used for detection: A, 5% ethanolic molybdatophosphoric acid for total lipids; B, ninhydrin reagent (Sigma) for aminolipids; C, zinzadze reagent (molybdenum blue spray reagent, 1.3%) for phospholipids; D, α-naphthol solution for glycolipids.

PE, phosphatidylethanolamine; APL, unidentified aminophospholipid, AL, unidentified aminolipid; L 1–7, unidentified lipids.
